# Engineered Bacteriophage as a Delivery Vehicle for Antibacterial Protein, SASP

**DOI:** 10.3390/ph14101038

**Published:** 2021-10-12

**Authors:** James Cass, Anne Barnard, Heather Fairhead

**Affiliations:** Phico Therapeutics, Bertarelli Building, Bourn Hall, Bourn, Cambridge CB23 2TN, UK; james.cass@phicotx.co.uk (J.C.); anne.barnard@phicotx.co.uk (A.B.)

**Keywords:** bacteriophage, phage, SASP, engineered phage, *Staphylococcus aureus*

## Abstract

The difficulties in developing novel classes of antibacterials is leading to a resurgence of interest in bacteriophages as therapeutic agents, and in particular engineered phages that can be optimally designed. Here, pre-clinical microbiology assessment is presented of a *Staphylococcus aureus* phage engineered to deliver a gene encoding an antibacterial small acid soluble spore protein (SASP) and further, rendered non-lytic to give product SASPject PT1.2. PT1.2 has been developed initially for nasal decolonisation of *S. aureus*, including methicillin-resistant *S. aureus*. Time-kill curve assays were conducted with PT1.2 against a range of staphylococcal species, and serial passaging experiments were conducted to investigate the potential for resistance to develop. SASPject PT1.2 demonstrates activity against 100% of 225 geographically diverse *S. aureus* isolates, exquisite specificity for *S. aureus*, and a rapid speed of kill. The kinetics of *S. aureus*/PT1.2 interaction is examined together with demonstrating that PT1.2 activity is unaffected by the presence of human serum albumin. SASPject PT1.2 shows a low propensity for resistance to develop with no consistent shift in sensitivity in *S. aureus* cells passaged for up to 42 days. SASPject PT1.2 shows promise as a novel first-in-class antibacterial agent and demonstrates potential for the SASPject platform.

## 1. Introduction

Given that the World Health Organisation (WHO) have stated that antibiotic resistance is one of the biggest threats to global health, new approaches to treating infectious diseases are clearly needed. In recent decades, commercial antibiotic development has frequently involved producing next-generations of existing classes of antibiotics, with novel classes being relatively few and far between [1].

Conventional antibiotics are generally broad spectrum in their activity, being unselective for the specific species of bacteria that is causing a particular infection. Whilst this is clearly valuable in treating patients prior to understanding what the causative pathogen is, it does have an impact on the composition of the microbiota, that is the indigenous microbes found ubiquitously in and on the human body, including in the gut and on skin [2]. Bacteriophages are ubiquitous with an estimated 10^31^ particles on the planet, and it seems highly probable that every bacterial species or strain will have phage that can infect it [3,4,5]. However, phages can be, and in fact most commonly are, exquisitely selective for their target species or strain of bacteria. This attribute opens up a broad scope for the applicability of phages in very diverse areas, including human and animal health, agriculture, and horticulture. Indeed, as medical understanding of the relevance to health of the microbiota increases, so phages are superbly placed to selectively manipulate the microbiota.

In fact, bacteriophages have a long and chequered history as antibacterial therapeutic agents [6,7,8]. However, as knowledge of bacteriophage biology, specificity, behaviour, and pharmacokinetics has increased, in parallel with genetic tools, so the possibility of engineering phages to optimise or change their characteristics for clinical application has become not just possible, but a reality [3]. This is enabling a new generation of commercially robust opportunities for phage-based antibacterials.

There are now a number of precision phage engineering methods which have been developed, including the Bacteriophage Recombineering with Electroporated DNA (BRED) method [9], the Host Range Determinant Selection (HORDS) method [10], cell-free transcription/translation (TX-TL) systems [3], in vitro assembly of an engineered phage genome from DNA fragments [3], and the use of yeast expression vectors to capture and engineer genomes prior to recovery of phages following transformation of a suitable bacterial host [3]. Most recently, and perhaps now the most widely adopted commercial approach, CRISPR-Cas-mediated phage engineering is being developed [11].

**Figure 1:**
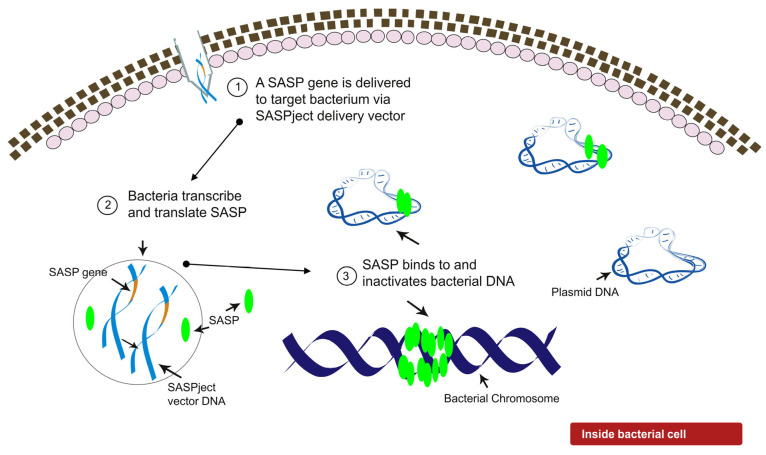
Mechanism of action of SASPject PT1.2 and SASP.

Phico Therapeutics’ concept for its SASPject technology, namely rationally designed engineering of bacteriophages as delivery vehicles for use in the treatment of bacterial infections, came to fruition in 2000. SASPject utilises small acid soluble spore proteins (SASPs) as the antibacterial agent with the gene encoding SASP delivered to target bacteria by engineered phage [12] (Figure 1). Spore forming bacteria produce α/β type SASPs and γ type SASPs [13]. The primary sequence of γ type SASPs has not been as highly conserved in evolution as has those of α/β type SASPs and they have no known function other than generating amino acids by their degradation during spore germination [13]. α/β type SASPs (hereinafter globally referred to as SASP(s)) comprise a small family of proteins that provide, or contribute to, protection of spore DNA from environmental stresses such as UV irradiation, heat, desiccation, and general temporal decay [14,15,16]. SASPs bind to and saturate the whole spore genome forming a SASP-DNA complex whereby a myriad of H-bonding between SASP and phosphate, ribose, and nucleobase groups, along the minor grove of DNA results in the conformation of bacterial DNA shifting from the normal B-like towards A-like [17]. In spores, SASP binding is protective, but an inability to degrade SASP prevents spore outgrowth and vegetative cell survival [18,19]. This natural and sequence-independent binding by SASPs means they can likely be considered inter-changeable with pan spectrum activity. It is also unlikely that resistance will develop to SASP activity since mutations in bacterial DNA do not affect the ability of SASP to bind to and inactivate DNA. Phico has developed a SASPject engineered phage for the intranasal decolonisation of *Staphylococcus aureus*, including MRSA (SASPject PT1.2).

This article focuses on the pre-clinical microbiology programme conducted with PT1.2, which provides early exemplification of the potential validity and specificity of the SASPject technology for the treatment of bacterial infections. Phico has taken PT1.2 into the clinic in a Phase 1 trial demonstrating a good safety and tolerability profile [20].

## 2. Results

### 2.1. Design of PT1.2

**Figure 2:**
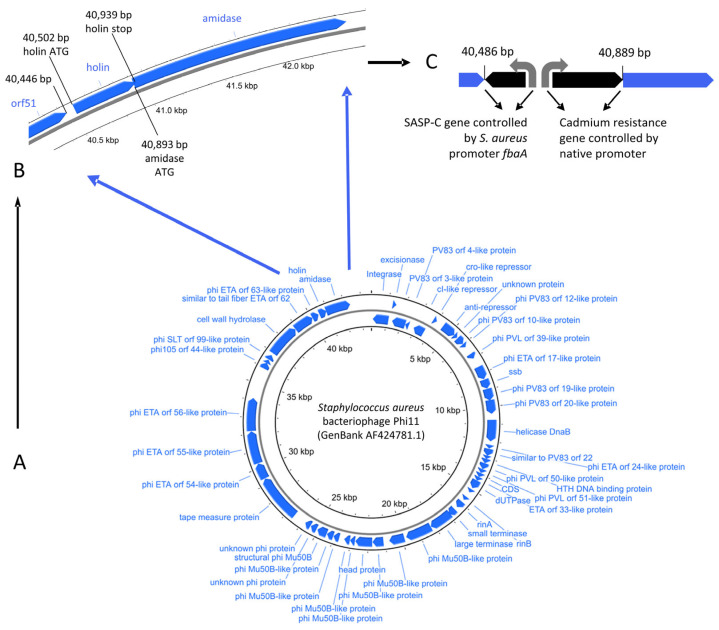
Modification of phi11 genome (**A**) showing holin and amidase genes (**B**) and creation of SASPject PT1.2 by deletion of holin gene and promoter driving holin and amidase genes by insertion of SASP-C gene under the control of native *S. aureus* promoter, *fbaA* and, selectable marker, cadmium resistance gene (**C**). Phi11 genome visualized using CGView [21].

PT1.2 was engineered using rational design techniques from the wild type temperate *S. aureus* phage, phi11. The phi11 phage was selected because the full genome sequence was available (Genbank AF424781.1), it had no apparent propensity to carry toxin genes, and it was tractable to manipulate. In 2000, when methods to manipulate and, more particularly, select, engineered lytic phage were limited, the use of a temperate phage was considered optimal. Engineering could be simply carried out with phi11 as a prophage and the modified phage could be maintained in a suitable *S. aureus* host so that it could be manufactured from a single master cell bank, rather than requiring an additional phage bank. *S. aureus* strain 8325-4 was selected as the final host strain for PT1.2 as it has been designated by the UK Health and Safety Executive as category 1, making it an ideal strain for manufacturing, particularly at large scale. Conventional cloning and allelic exchange techniques were employed to rationally design the engineered PT1.2, with positive selection for modified phi11 enabled by insertion of a gene encoding cadmium resistance downstream of the SASP gene and selection on CdCl_2_ agar plates. The SASP-C gene from *Bacillus megaterium* was used in conjunction with a constitutive *S. aureus* promoter, *fbaA.* This SASP expression cassette replaced the phi11 native holin gene and was inserted in the opposite orientation to the phi11 late genes. Deletion of the holin gene and thus eliminating the presence of holin within target bacteria means that the phage endolysins cannot access the bacterial cell wall and cause lysis. Inhibiting the ability of PT1.2 to lyse targeted *S. aureus* bacteria was considered desirable since it enabled production of SASP to continue within targeted cells and also inhibited the release of any toxins or toxin/antibiotic resistance genes into the infection environment. Similarly, prevention of lysis minimised the release of inflammatory *S. aureus* cell wall components that are caused by phage-mediated lysis. The extended production time of SASP was designed to maximise its coverage of the target *S. aureus* genome and plasmid DNA: preliminary data suggests SASP-bound plasmid DNA is not taken up by other cells, which may help to limit the spread of plasmid-borne antibiotic resistance genes (data not shown). The development of PT1.2 is shown in Figure 2.

### 2.2. Spectrum of Activity and Specificity of PT.2

All 225 isolates of *S. aureus* tested showed susceptibility to SASPject PT1.2 using a range of methods comprising kill curves, or no growth of targeted cells 3 h after exposure to PT1.2 in microtitre plate assays (Table 1). In the 3 h kill assay 153/163 (93.7%) MRSA and 23/34 (67.6%) MSSA isolates showed a 4 log_10_ or greater kill (a drop from 10^5^ cfu/mL to 10 or fewer cfu/mL), with no viable cells observed at 3 h [21]. In time-kill curves, at the 4 h time point there were 15/24 (63%) MRSA and 2/4 (50%) MSSA isolates showing a 4 log_10_ or greater kill. Overall 177/187 (94.6%) MRSA and 29/38 (76.3%) MSSA isolates showed a ≥3 log_10_ kill by 3 or 4 h.

Among the 10 isolates of MRSA where a less than 3 log_10_ reduction in viable count was achieved, 4 strains (7079, 88032, 17047 from the UK and 7050 from the USA) had no further distinguishing details, whilst the other 6 strains (160006, 100096 from the UK, 160002 from Japan and strains 14545, 15818, 24764 of unknown geographical origin) exhibited intermediate resistance to vancomycin. All 12 Panton-Valentine Leukocidin

(PVL)-positive MRSA and MSSA strains exhibited a 4 log_10_ or greater kill within 3 h.

In the specificity studies none of 24 tested and confirmed coagulase-negative staphylococci showed any reduction in cell counts, confirming PT1.2 specificity (Table 2).

**Figure 3:**
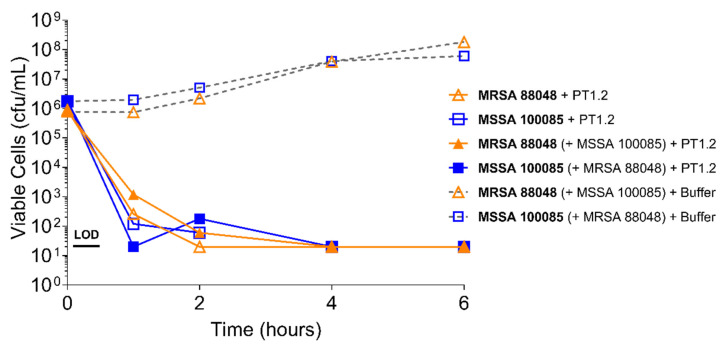
Time-kill curve assay showing efficacy of SASPject PT1.2 in mixed *S. aureus* MSSA/MRSA (strains 100085/88048 respectively) cultures. Viable cells counts are shown for the strain written in bold letters. Results of a representative experiment are shown.

**Figure 4:**
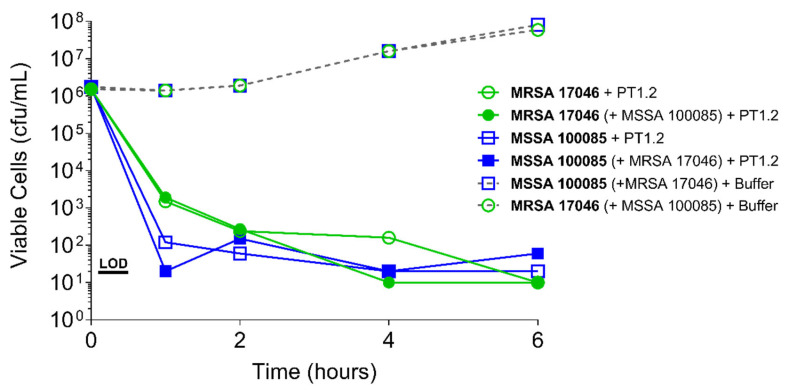
Time-kill curve assay showing efficacy of SASPject PT1.2 in mixed *S. aureus* MSSA/MRSA (strains 100085/17046 respectively) cultures. Viable cells counts are shown for the strain written in bold letters. Results of a representative experiment are shown.

**Figure 5:**
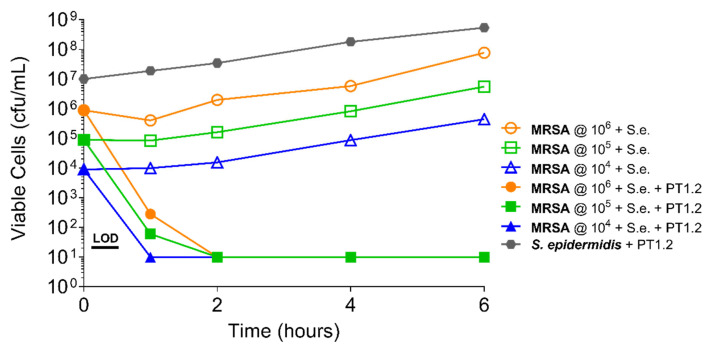
Time-kill curve assay showing efficacy of SASPject PT1.2 in mixed cultures of MRSA strain 88048 and *S. epidermidis* 7,020 (abbreviated to “S.e.” in the chart legend). Viable cell counts are shown for the strain written in bold letters. Results of a representative experiment are shown.

**Figure 6:**
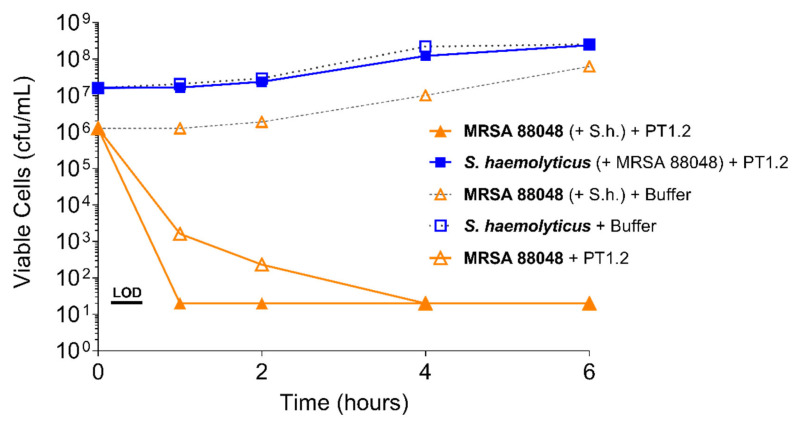
Time-kill curve assay showing efficacy of SASPject PT1.2 in mixed cultures of MRSA and *S. haemolyticus* 133034 (abbreviated to “S.h.” in the chart legend). Viable cell counts are shown for the strain written in bold letters. Results of a representative experiment are shown.

PT1.2 was equally effective against both MRSA strain 88048 and MSSA strain 100085 in mixed culture (Figure 3), as well as MRSA strain 17046 and MSSA strain 100085 in mixed culture (Figure 4), showing a 4 log_10_ or greater reduction in cell viability within 2 or 3 h, respectively. Activity against these strains in co-culture did not differ materially from the activity of PT1.2 against each strain alone although the rate of kill of the MSSA strain in co-culture was slightly faster than for either MRSA strain. Likewise the presence of *S. epidermidis* strain 72020 at 10^7^ cfu/mL in co-culture with MRSA 88048 at cell concentrations ranging from 10^4^–10^6^ cfu/mL had no effect on activity of PT1.2 (Figure 5). A > 4 log_10_ drop in cell viability was seen for all MRSA 88048 cultures (both alone and in co-culture) within 2 h of addition of PT1.2. A co-culture of *S. haemolyticus* strain 133034 (10^7^ cfu/mL) with MRSA strain 88048 (10^6^ cfu/mL) also had no effect on PT1.2 activity against the MRSA (Figure 6).

### 2.3. Speed of Kill

**Figure 7:**
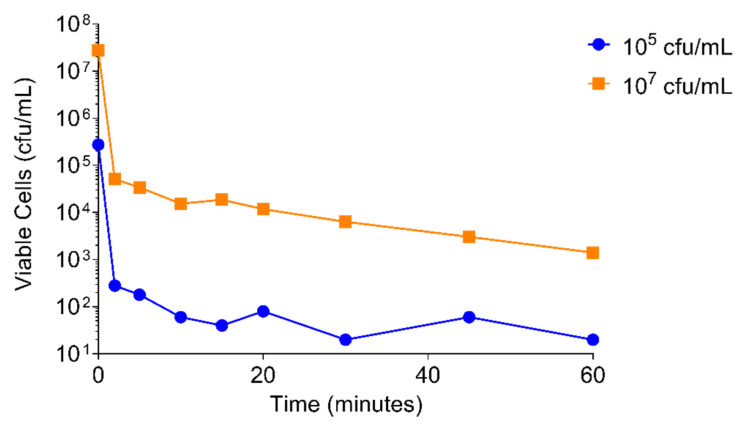
Time-kill curve assay of SASPject PT1.2 activity against *S. aureus* USA 300 over 1 h. Results of a representative experiment are shown.

A 3 log_10_ drop in viability of *S. aureus* USA300 was achieved within 2 min of adding PT1.2 to a culture of 10^5^ cfu/mL and within 10 min of addition to a culture of 10^7^ cfu/mL (Figure 7).

### 2.4. Kinetics of PT1.2:S. aureus Interaction

#### 2.4.1. PT1.2 Concentration

**Figure 8:**
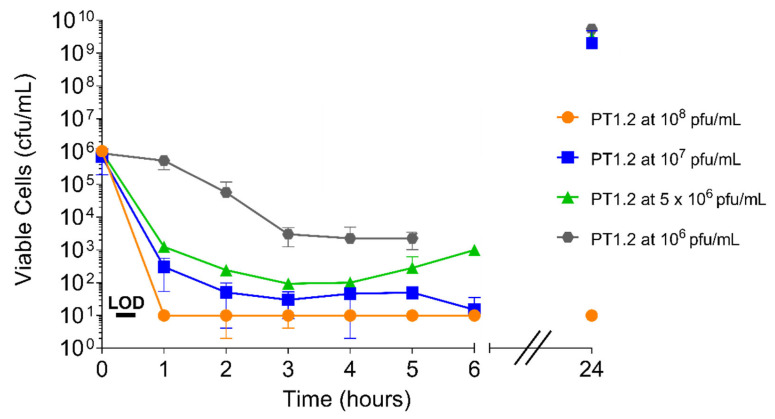
Time-kill curve analysis of SASPject PT1.2 activity, at concentrations ranging 10^6^ to 10^8^ pfu/mL, against EMRSA-15 strain 02ST4127 treated over 24 h. Data plotted are the means of 2 or 3 (10^7^ pfu/mL only) replicates. Error bars represent 1 standard deviation.

Both the speed and extent of activity of PT1.2 against *S. aureus* 02ST4127 were affected by PT1.2 concentration (Figure 8). A reduction in viable cell numbers to below the limit of detection (10 cfu/mL) within 2 h with PT1.2 at 10^8^ pfu/mL was consistent for all triplicate cultures. However, there was marked variation in the speed and extent of kill between triplicate cultures with all other concentrations of PT1.2. For all concentrations except 10^8^ pfu/mL, where viable cell numbers remained below the limit of detection, there was varied regrowth of surviving cells at 24 h.

#### 2.4.2. *S. aureus* Concentration

**Figure 9:**
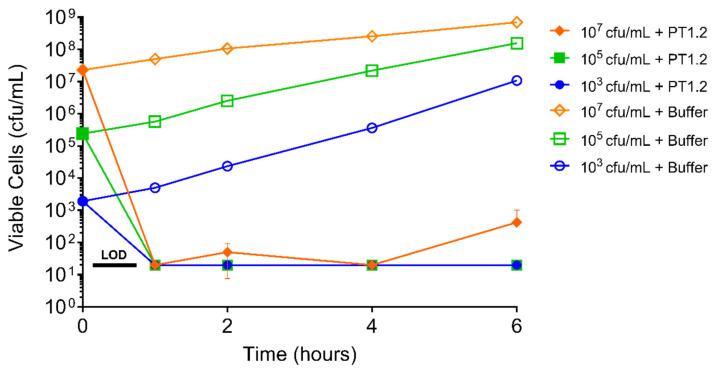
Time-kill curve analysis of SASPject PT1.2 activity against EMRSA-15 strain 02ST4127, at viable cell concentrations of 10^3^, 10^5^, and 10^7^ cfu/mL, over 6 h. The means of duplicate samples are plotted. Error bars represent 1 standard deviation.

PT1.2 exhibited a rapid kill against *S. aureus* USA300 at concentrations spanning 1.5 × 10^3^ to 1.5 × 10^7^ cfu/mL showing a drop in viable cell numbers to below the limit of detection (20 cfu/mL) within 1 h for all cultures (Figure 9).

### 2.5. Effect of Human Serum Albumin on PT1.2 Activity

**Figure 10:**
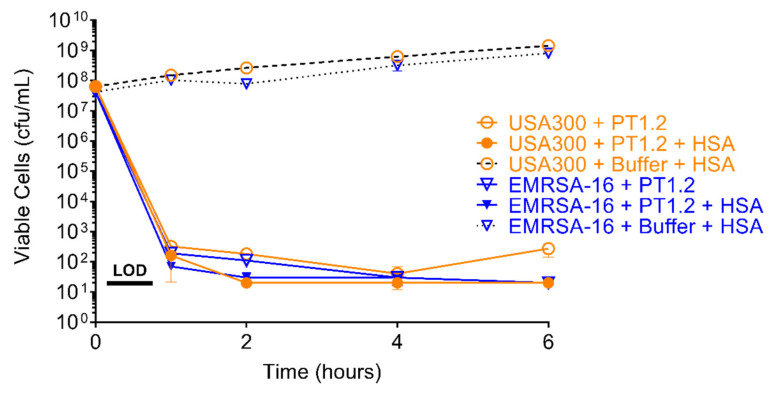
Time-kill curve assay showing the efficacy of SASPject PT1.2 against *S. aureus* MRSA USA300 and EMRSA-16 in the presence and absence of human serum albumin (HSA) at 50 g/L. Data points represent the mean of 2 replicates and error bars represent 1 standard deviation.

The activity of PT1.2 against both *S. aureus* USA300 and EMRSA-16 was not compromised by the presence of HSA (50 g/L) (Figure 10). A 5-log_10_ or greater reduction in viable cell numbers was observed within 1 h in the presence and absence of HSA.

### 2.6. Assessment of S. aureus Resistance to PT1.2

**Figure 11:**
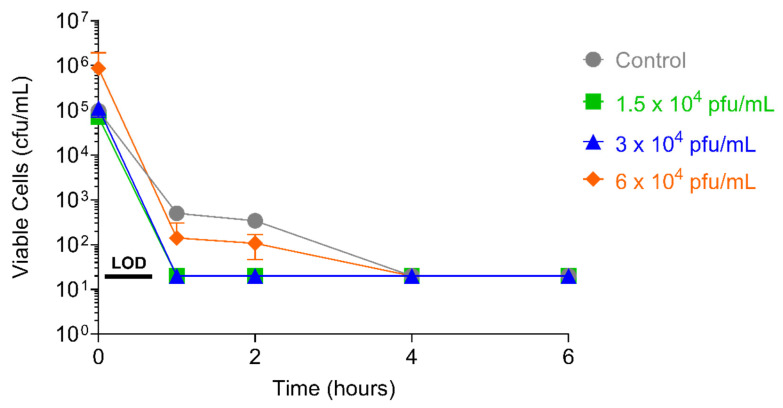
Assessment of resistance development in serially passaged EMRSA strain O2ST4285 by time-kill curve assay of cells from passage day 52 (3 and 6 × 10^4^ pfu/mL passaged cultures) and passage day 46 (1.5 × 10^4^ pfu/mL passaged cultures) exposed to SASPject PT1.2. Passaging was carried out in triplicate, and time-kill curve assays were carried out in singlicate on each of the triplicate cultures. Cells that were passaged in singlicate in the absence of PT1.2 for 52 days were exposed to PT1.2 as a control (Control). The mean number of viable cells for each passaging condition following time-kill curve assay with PT1.2 is plotted. Error bars for the passaged conditions represent 1 standard deviation.

**Figure 12:**
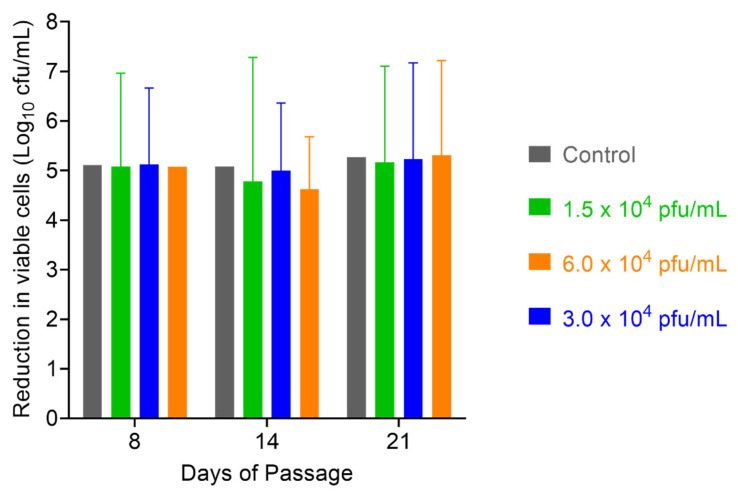
Assessment of resistance development in serially passaged MRSA USA300 by exposing passaged cells to PT1.2 at passage days 7, 14, and 21, and incubating with PT1.2 for 4 h before assessing viable cell count. Triplicate cultures were set up for each of the phage concentrations, and each culture was assessed via the kill experiment in singlicate. Results shown are the mean viable cell counts for the three cultures passaged with each PT1.2 concentration, following kill experiment analysis. Error bars represent 1 standard deviation. The control was MRSA USA300 simultaneously passaged for the same lengths of time in the absence of PT1.2, prior to assessment of viable cell count after exposure to PT1.2 for 4 h.

After 52 days of serial passaging, when the study was terminated (46 days for the 1 × 10^4^ pfu/mL dose of PT1.2), the susceptibility of EMRSA strain O2ST4285 to PT1.2 remained equal to that observed for the control (Figure 11). For *S. aureus* USA300 no consistent or significant shift in susceptibility to PT1.2 was seen during passaging over the 21 days of the experiment (Figure 12). In the single dosing study, no PT1.2-resistant isolates of *S. aureus* USA300 were found amongst the 1.2 × 10^8^ cfu tested.

## 3. Discussion

Phage therapy has been undergoing a resurgence in recent years driven, at least in part, by the “next generation phage therapy” that comprises engineered phage. Engineering can facilitate precision control of important considerations such as host range, manufacturability, and PK profile, for example, by transferring the genetics of these traits at optimum into a single or small group of phages making up a drug product. Engineering enables a move away from the large cocktails of diverse phages traditionally used in wild type phage therapy by concentrating these attributes across much fewer, ideally related, phages.

Phico engineers phages to deliver a gene encoding a small acid soluble spore protein, SASP, to target bacterial species. The SASP gene is placed under the control of a constitutive promoter from the target bacterium so that, following injection of the SASP gene, intracellular expression is immediate and continual. SASPs bind in a non-sequence specific manner via two highly conserved regions (AIDQMKYEIASEFGVNLG and TSRANGSVGGEITKRLVQM) resulting in a shift in DNA conformation from the normal B-like to A-like [16,17]. The amino acid sequences of SASPs show exceptional conservation both within and across species of endospore-forming bacteria, both aerobic and anaerobic, yet they show minimal homology to bacterial gene sequences in current databases [16]. Uniquely, in view of their mode of action, SASP also bear no sequence similarity to other DNA-binding proteins in available databases and contain no known sequence motifs, including motifs found in other DNA-binding proteins [16]. Expressed in vegetative bacteria, SASPs act to inactivate the bacterial DNA by preventing DNA replication and, where bound, transcription.

SASPject PT1.2 comprises a single temperate phage in which the holin gene has been removed and replaced with a SASP gene expression cassette containing the SASP-C gene from *Bacillus megaterium* under the control of constitutive *S. aureus* promoter, *fbaA*. PT1.2 is derived from *S. aureus* phage, ϕ11. PT1.2 has been developed for the intranasal decolonisation of *S. aureus*, including MRSA and is in further pre-clinical development for systemic applications. Given that 33% of surgical site infections (SSIs) can be caused by *S. aureus* [22], evidence that nasal *S. aureus* decolonisation can lead to a decreased incidence of *S. aureus* infection has led to this practice being implemented on a wide scale, and recommended, in conjunction with chlorhexidine body washing, by the UK National Institute for Health and Care Excellence [23]. There remain limited options for effective nasal decolonisation of *S. aureus*; mupirocin is the primary agent of choice although resistance rates of up to 9% have been reported [24]. The use of other antibiotics is also potentially impacting on mupirocin efficacy with a study showing that sub-MIC levels of ciprofloxacin increase adhesion of quinolone-resistant MRSA, perhaps explaining persistent MRSA colonization and failure of mupirocin treatment in patients who received a fluoroquinolone [25,26]. That mupirocin remains the most effective antibiotic for MSSA and MRSA decolonisation, both in patients and healthcare personnel, means a reduction of its effectiveness presents a risk for invasive infection [24]. Hence, there remains a need for a new topical agent with potent efficacy, ideally bactericidal, and a low propensity for resistance development.

SASPject PT1.2, targeted at *S. aureus*, including MRSA has undergone a programme of pre-clinical microbiology testing that supported testing of PT1.2 in a Phase 1 clinical trial. PT1.2 shows rapid bactericidal activity against a wide range of geographically and genetically diverse *S. aureus*, including strains that are methicillin resistant and susceptible, vancomycin intermediate and heterologous vancomycin intermediate resistant, and both Panton-Valentine Leukocidin positive and negative [27,28,29,30]. Indeed, almost 95% (177/187) of MRSA and just over 76% (29/38) of MSSA isolates exhibited at least a 3 log_10_ kill within 4 h of addition of PT1.2. Remaining isolates were susceptible to SASPject PT1.2, but the killing rates were less impressive. No activity was evident against 24 coagulase-negative staphylococci representing nine species, confirming a high degree of target pathogen specificity. Many antibacterial agents, including existing “narrow spectrum” conventional agents will eliminate a significant proportion of the patient’s normal microbiota, leaving the body more susceptible to colonisation or infection by pathogens such *Neisseria meningitidis* or *Streptococcus pneumoniae,* for example, in the nose. As SASPject technology can be specifically targeted at selected pathogens, its use should have a minimal effect on the body’s normal bacterial microbiota and may result in fewer secondary infections or the selection of resistant colonising bacteria.

A key feature of the antibacterial component of SASPject, SASP, is the rapidity of its activity against *S. aureus*. Typically, for conventional antibiotics, rate of kill studies involve sampling at hourly intervals seeking to establish a 3 log_10_ reduction in viable cell count over 24 h [31]. SASP can achieve up to a 3 log_10_ drop in cell viability within 10 min in a 10^7^ cfu/mL culture, and a 6 log_10_ reduction within 1 h against actively growing cells [24]. SASP is also rapidly bactericidal against stationary phase *S. aureus* (data not shown).

As the delivery of the SASP gene by SASPject delivery vectors is a truly novel approach to antibacterial therapy, it is expected to be unaffected by existing antibiotic resistance mechanisms. Indeed, in addition to the data given above for methicillin and intermediate vancomycin resistance, SASPject PT1.2 is minimally affected by all tested resistance mechanisms: penicillin, tetracycline, ciprofloxacin, rifampicin, erythromycin, fusidic acid, and gentamycin [23]. The rate of kill against some strains, including some, but not all, VISA strains, can show more variation and be generally slower. However, the mechanism for this is not clear and warrants further investigation.

That PT1.2 activity is unaffected by the presence of human serum albumin is encouraging and suggestive that protein:protein interactions in the body will not impact efficacy; the absence of any measurable binding to HSA is also positive for future systemic applications.

Due to the unique mode of action of SASP-binding to bacterial DNA irrespective of sequence, the likelihood of resistance developing to SASP is judged to be very low. Germinating spores clearly need to release their genome from SASP in order to outgrow. SASP degradation during spore germination is initiated by a single protease, GPR [32,33,34]. This enzyme is an endoprotease specific for a pentapeptide sequence found once or twice in all SASP. GPR is initially synthesized during sporulation and is processed twice to create a 40-kDa form, which then acts to initiate SASP degradation in vivo. Crucially, the activity of GPR is dependent on the conditions inside a germinating spore, i.e., low water activity, low pH, and the presence of dipicolinic acid, collectively, conditions that are not possible in vegetative bacteria [35]. However, the possibility of resistance arising to the SASPject phage to prevent injection of the SASP gene into target bacteria must also to be considered. Resistance to wild type viral vectors is a widely acknowledged phenomenon. However, the presence of a constitutively expressed SASP, which remains active independently of the fate of the phage delivery vehicle inside target cells, renders ineffective all of the intracellular resistance mechanisms that bacteria use against wild type viral vectors. A remaining resistance mechanism occurs at the stage of phage vector binding to the bacterial cell surface.

It has been suggested that staphylococcal phages bind to a common, and historically highly conserved, cell wall receptor [36,37]. This suggestion has been supported by the studies reported here, showing PT1.2 activity against all tested *S. aureus* strains. Furthermore, serial passaging studies of between 46 and 52 days, with 9 cultures of *S. aureus* strain O2ST4285 (EMRSA-16) and PT1.2 at inhibitory and sub-inhibitory levels, showed no consistent trend towards resistance to phage binding [38]. No naturally occurring resistance was observed in the >1 × 10^8^ tested *S. aureus* cells. That the limiting factor deciding host spectrum of *S. aureus* phages occurs post-injection of phage vector DNA has been borne out by studies, confirming that SASPject PT1.2 is effective against isolates (including EMRSA-15 types), which do not support the parental wild type viral vector (data not shown).

A theme in some of the kill curve studies reported here is that of occasional regrowth of target cells to highly variable levels. Since remaining viable cells do not exhibit resistance when used in further time-kill assays, one or more other factors are influencing the outcome. Compared to the number of molecules of a conventional antibiotic, even at sub-MIC level, very few phage particles are used. As such, limited physical interaction between phage vector and bacteria is likely to be a contributory factor and this phenomenon is currently being investigated further.

Overall, important considerations for a commercial antibacterial agent using bacteriophage-based technologies include host range, manufacturability, pharmacokinetics, and stability. All, with perhaps the exception of stability, have the potential to be improved by engineering of phages. Host range can be broadened by engineering phages that express a multiplicity of host binding domains to steer away from extensive phage cocktails and limit the number of phages that make up a product, or engineering different versions of a single lead phage each expressing a modified or alternative host binding domain [39,40,41,42]. Limiting the number of phages in a product simplifies the issue of pharmacokinetics where different phages can have widely varying half lives in the body making judgements about dosing intervals/regimens difficult; again, this has potential to be controlled/improved by phage engineering [43]. For manufacturing, the fewer phages per product, the better, as each phage can have its own production and purification requirements, making the process to a drug product more challenging to control and show robustness and reproducibility. In addition, the final step of mixing the phages to make the product, in exact, consistent ratios whilst maintaining sterility, introduces greater risk as the number of different phages increases. Missteps at this stage can lead to wastage of batches driving up cost of goods. Thus, phage engineering, with the promise of limiting phage numbers, can have a major impact on simplifying manufacturing. Stability is a key consideration, and the importance of this was demonstrated in a phage therapy clinical trial for the topical treatment of *P. aeruginosa* infected burns [44]. This so-called Phagoburn study involved treatment with cocktails of up to twelve *P. aeruginosa* phages at a planned dose of 10^6^ pfu/mL. The study was halted prematurely due to failure to show activity, and it was subsequently found that the active titre of the phage preparation had dropped to 10^1^–10^2^ pfu/mL.

Commercially, phage therapy remains in its infancy with infrequent compassionate-use cases [45,46,47]. To become a more widespread treatment option, well-designed clinical trials, using phages that are the product of robust, reproducible, well-controlled manufacturing processes, need to be executed with both engineered and wild type phages.

## 4. Materials and Methods

### 4.1. The Strains, Media and Chemicals

MRSA USA300 strain 43484 and EMRSA-16 strain 252 were routinely used for time-kill curves, except where stated otherwise.

Overnight cultures were grown in Luria-Bertani (LB) broth (5 mL) in sterile Universal tubes, incubated at 37 °C with shaking (350 rpm) and diluted for time-kill curves in LB broth containing CaCl_2_ (10 mM), pH 7.2 (LBC broth). SASPject PT1.2 suspended in Tris buffered saline (50 mM) containing CaCl_2_ (10 mM) and MgCl_2_ (1 mM) (ΦTBS) was used at a standard, final concentration of 1.5 × 10^8^ pfu/mL, unless stated otherwise.

Controls were performed for each experiment where PT1.2 was substituted with an equivalent volume of ΦTBS.

### 4.2. Spectrum of Activity of PT1.2

#### 4.2.1. 3 h Kill Assay

See Table 1 for characteristics of *S. aureus* isolates. Cultures were grown to mid-8 log_10_ phase and diluted to ~2 × 10^5^ cfu/mL in LBC broth. Diluted culture (55 μL) was added to PT1.2 (55 μL) in a microtitre well. Growth controls for each strain were set up where PT1.2 was replaced with ΦTBS. Aliquots of 100 μL were removed from each sample well after 3 h incubation at 35 °C and spread onto blood agar plates. Colony counts were carried out after overnight incubation at 35 °C.

#### 4.2.2. Time-Kill Curves

Cultures (see Table 1 for isolate characteristics) were grown overnight and diluted to a final inoculum of 10^5^ cfu/mL with reference to a McFarland standard. PT1.2 was added to the diluted cultures. Tubes were incubated at 35–37 °C without shaking and aliquots (100 μL) were removed at 0, 1, 2, 4, 6, and 24 h and spiral plated (50 μL) onto nutrient agar plates or blood agar plates. Colony counts were carried out after overnight incubation at 35–37 °C.

### 4.3. Specificity of PT1.2

#### 4.3.1. Activity of PT1.2 against Coagulase Negative Staphylococci

The 3 h kill assay was also conducted with twenty four isolates (Health Protection Agency (HPA) Cambridge identity codes (CC) given) of the staphylococcal species listed in Table 3.

#### 4.3.2. Activity of PT1.2 in Mixed Bacterial Cultures

Isolates used for mixed culture kill assays are listed in Table 4.

MSSA/MRSA co-cultures: MSSA/MRSA cultures were diluted to a final inoculum of 4 × 10^6^ cfu/mL. In Universal tubes, 200 μL of MSSA culture was mixed with an equal volume of MRSA culture and 400 μL of PT1.2 was added, yielding final concentrations of 10^6^ cfu/mL for each bacterial strain and 1.5 × 10^8^ pfu/mL of PT1.2. In addition to a ϕTBS control, LBC broth (200 μL) was added to a mono-culture of each strain (200 μL) plus PT1.2 (200 μL).

MRSE/MRSA co-culture: MRSE was diluted to 4 × 10^7^ cfu/mL from an overnight culture, and MRSA strain 88,048 was diluted to 4 × 10^6^, 4 × 10^5^ and 4 × 10^4^ cfu/mL from an overnight culture. In Universal tubes, 200 μL of diluted MRSE culture was mixed with an equal volume of MRSA culture and 400 μL PT1.2 was added. This yielded final concentrations of 10^7^ cfu/mL MRSE with either 10^6^, 10^5^, or 10^4^ cfu/mL MRSA and 1.5 × 10^8^ pfu/mL of PT1.2. In addition to a ϕTBS control, MRSE was replaced by an equal volume of LBC broth (200 μL) to give MRSA only.

MRSH/MRSA co-cultures: Overnight cultures of MRSH and MRSA strain CC 88,048 were diluted to 4 × 10^7^ cfu/mL and 4 × 10^6^ cfu/mL, respectively. In Universal tubes, 200 μL of diluted MRSH culture was mixed with an equal volume of MRSA culture and 400 μL PT1.2 was added. This yielded final concentrations of 10^7^ cfu/mL MRSH, 10^6^ cfu/mL MRSA and 1.5 × 10^8^ pfu/mL of PT1.2. In addition to a ϕTBS control, MRSH was replaced by an equal volume of LBC broth (200 μL) to give MRSA only.

All samples were incubated at 35 °C without shaking and aliquots (150 μL) were removed at 0, 1, 2, 4, and 6 h and spiral plated (50 μL) onto both Colombia blood agar plates and chromID MRSA plates (Biomerieux). Colony counts were carried out after overnight incubation at 35 °C with total cell numbers enumerated on Colombia blood agar plates and MRSA cells enumerated on chromID plates. The number of non-MRSA surviving colonies was calculated by subtracting the cfu/mL derived from each chromID MRSA plate from the number derived from the corresponding Colombia blood agar plate.

### 4.4. Effect of PT1.2 on Bacterial Viability

#### 4.4.1. Speed of Kill

*S. aureus* MRSA USA300 was grown overnight at 37 °C without shaking, and then diluted to a final concentration of either 2.7 × 10^7^ or 2.7 × 10^5^ cfu/mL. PT1.2 was added to each culture dilution. Cultures were incubated at room temperature without shaking and aliquots (100 μL) were removed at 0, 2, 5, 10, 15, 20, 30, 45 and 60 min and spiral plated (50 μL) onto blood agar plates. Colony counts were enumerated after overnight incubation at 37 °C.

#### 4.4.2. Concentration of PT1.2

An overnight culture of EMRSA-15 strain 02ST4127 was diluted to 4 × 10^6^ cfu/mL and aliquots mixed with an equal volume of PT1.2 to give final concentrations of 10^8^, 10^7^, 5 × 10^6^ and 10^6^ pfu/mL. Cultures were incubated at 37 °C without shaking and aliquots (100 μL) were removed at 0, 1, 2, 3, 4, 5, 6, and 24 h and plated (100 μL) onto LB agar plates. Colony counts were enumerated after overnight incubation at 37 °C.

#### 4.4.3. Concentration of Bacterial Culture

Time-kill curves were carried out with PT1.2 added to overnight cultures of *S. aureus* EMRSA-15 strain O2ST4127 each diluted giving final concentrations of 1.5 × 10^7^, 1.5 × 10^5^ and 1.5 × 10^3^ cfu/mL. Cultures were incubated at 37 °C without shaking and aliquots (100 μL) were removed at 0, 1, 2, 4 and 6 h and spiral plated (50 μL) onto LB agar plates. Colony counts were enumerated after overnight incubation at 37 °C.

### 4.5. Effect of Human Serum Albumin (HSA) on PT1.2 Activity

*S. aureus* USA300 and EMRSA-16 were grown overnight and then each diluted to 2 × 10^7^ cfu/mL. PT1.2 was added to 2 × 10^7^ pfu/mL (final concentration) and after removing a T0 (100 μL) aliquot, HSA was added to a final concentration of 50 g/L. The samples plus controls were incubated without shaking at 37 °C, and aliquots (60 μL) removed at 1, 2, 4, and 6 h. All aliquots were spiral plated (50 μL) on LB agar plates. Colony counts were performed after overnight incubation at 37 °C.

### 4.6. Assessment of S. aureus Resistance to SASPject PT1.2

#### 4.6.1. Passaging Studies

An EMRSA-16 strain O2ST4285 culture was grown overnight at 37 °C. The culture was diluted to 10^5^ cfu/mL (final concentration) and PT1.2 was added to final, sub-inhibitory concentrations of 1.5 × 10^4^, 3 × 10^4^ or 6 × 10^4^ pfu/mL, each in triplicate. Triplicate controls, with an equal volume of LBC broth replacing PT1.2, were also set up. Cultures were grown overnight at 37 °C without shaking. Daily for 52 successive days (46 days for cultures with 1.5 × 10^4^ pfu/mL PT1.2) aliquots of sample and control cultures were diluted to 10^5^ cfu/mL, PT1.2 was added as described above, and again grown overnight. The susceptibility of the cultures to PT1.2 was assessed by time-kill assay at weekly intervals: 10^5^ cfu/mL of each passaged culture and 1.5 × 10^8^ pfu/mL PT1.2 were mixed and incubated at 37 °C without shaking. Aliquots (75 µL) were removed at 1, 2, 4, and 6 h and spiral plated (50 µL) onto LB agar plates and colonies enumerated after overnight incubation at 37 °C. This study was repeated with *S. aureus* USA300 for a period of 21 days except susceptibility to PT1.2 was assessed on days 7, 14, and 21 at a single 4 h time point.

#### 4.6.2. Single Dosing Study

In order to estimate the frequency of any naturally occurring resistance to PT1.2, a culture of *S. aureus* USA300 (1.2 × 10^7^ cfu/mL) was aliquoted into 10 × 1 mL fractions and centrifuged. Each pellet was resuspended in LBC broth containing PT1.2 (1.5 × 10^8^ pfu) and incubated for 4 h at 37 °C, and then spread on to LB agar plates and incubated overnight at 37 °C. Any colonies which grew were re-tested for susceptibility to PT1.2 by time-kill curve as described above.

## 5. Conclusions

In summary, SASPject PT1.2 has provided promising in vitro data against *S. aureus* to support its further development as well as demonstrating the potential of SASPject as a novel antibacterial platform technology. Given the expected pan spectrum antibacterial activity of SASP, coupled with the ubiquity of phages for a target bacterial species, SASPject technology has the potential to be directed against any selected Gram positive or Gram negative bacterial target. SASPject PT1.2 demonstrates rapid bactericidal activity and is effective against all of the diverse range of *S. aureus* isolates tested, whilst showing complete specificity with activity unaffected by the presence of other staphylococcal species. SASPject PT1.2 showed a low propensity for resistance to develop. SASPject PT1.2 and the SASPject platform has the potential to provide a new therapeutic approach and a novel class of biological antibacterial that can be selectively targeted to individual or multiple bacterial species or genera.

## Figures and Tables

**Table 1:** Details of methicillin resistant *S. aureus* (MRSA) isolates (*n* = 187) and methicillin sensitive *S. aureus* (MSSA) isolates (*n* = 38) with log_10_ drop in viability at 3 h (3 h kill assay) or 4 h (time-kill curves).

Character	Detail (Total No. of Isolates, *n* = 225) (Total No. of Isolates in the 3 h (3 h) or Time-Kill (tk) Assays)	No. of Isolates					
		3 h Kill Assay log_10_ Drop at 3 h			Time-Kill Curves log_10_ Drop at 4 h		
		≥2	≥3	≥4	≥2	≥3	≥4
*mecA*	*mecA*^−^ (38) (3 h = 34; tk = 4)	26	26	23	3	3	2
	*mecA*^+^ (187) (3 h = 163; tk = 24)	158	156	153	22	21	15
Panton Valentine Leukocidin (PVL)	PVL^−^ (3 h = 10)	10	10	10			
	PVL^+^ (3 h = 12)	12	12	12			
SCC*mec*	I (3 h = 22)	21	21	20			
	II (3 h = 31)	31	31	31			
	III (3 h = 23)	23	23	23			
	IV (3 h = 38)	37	36	36			
	V (3 h = 1)	1	1	1			
	Untypeable (3 h = 3)	3	3	3			
Sequence type	1 (3 h = 3)	3	3	3			
	22 (3 h = 15)	15	15	15			
	30 (3 h = 1)	1	1	1			
	36 (3 h = 12)	12	12	12			
	80 (3 h = 2)	2	2	2			
	239 (3 h = 6)	6	6	6			
	240 (3 h = 2)	2	2	2			
	247 (3 h = 4)	4	4	4			
	5 (3 h = 6)	6	5	5			
	8 (3 h = 16)	16	15	15			
USA type	ORSA-I (3 h = 7)	7	7	7			
	ORSA-II (3 h = 13)	13	12	12			
	ORSA-III (3 h = 7)	7	7	7			
	ORSA-IV (3 h = 14)	14	14	14			
	ORSA-I/III/IV (3 h = 11)	11	10	10			
	USA-100 (3 h = 1; tk = 1)	1	0	0	1	1	1
	USA-200 (13), -300 (1), -400 (1), -500 (1), -700 (1), -800 (1), -1000 (1), -1100 (1) (3 h = 20)	12	12	12			
VISA	VISA (3 h = 3; tk = 3)	2	0	0	2	2	0
hVISA	hVISA (3 h = 9; tk = 2)	9	9	0	1	0	0
Historic Clones	Archaic/Iberian (3 h = 15)	15	14	14			
	Brazilian/Hungarian (3 h = 28)	28	28	28			
	Pediatric (3 h = 6)	6	6	6			
UK epidemic clones	EMRSA-1 (1), -2 (1), -3 (3), -4 (3), -5 (2), -6 (3), -7 (2), -8 (2), -9 (2), -10 (2), -11 (2), -12 (3) -13 (2), -14 (1), -15 (14), -16 (11), -17 (3) (3 h = 57)	54	54	54			
	EMRSA-6 (3 h = 3)	3	3	2			
Community MRSA	(3 h = 10; tk = 5)	10	10	10	5	5	5
Geographic location	Australia (3 h = 6)	6	6	6			
	Japan (3 h = 4)	4	3	3			
	USA (3 h = 16)	15	15	15			
	Denmark (3 h = 1)	1	1	1			
	France (3 h = 2)	2	2	2			
	Ireland (3 h = 5)	5	5	5			

**Table 2:** Log_10_ reduction in viable cells of coagulase negative Staphylococcal species and strains tested in 3 h kill assays.

Staphylococcal Species	Cambridge Identity Codes (CC)	Log_10_ Reduction in Viable Cells
*S. epidermidis*	72003	0
	72037	0
	72025	0
	72004	0
	72029	0
	72020	0
	72030	0
*S. haemolyticus*	133072	0
	133034	0
	133068	0
*S. warneri*	133029	0
	133019	0
*S. hominis*	133075	0
	133097	0
*S. cohnii*	133089	0
	133041	0
*S. capitis*	133095	0
	133092	0
*S. simulans*	133080	0
	133053	0
*S. lugdunensis*	133074	0
	133082	0
*S. saprophyticus*	133002	0
	133091	0

**Table 3:** List of coagulase negative Staphylococcal species and strains tested for sensitivity against PT1.2 in 3 h kill assays.

Staphylococcal Species	Cambridge Identity Codes (CC)	Methicillin Sensitivity Profile
*S. epidermidis*	72003	S
	72037	S
	72025	S
	72004	R
	72029	R
	72020	R
	72030	R
*S. haemolyticus*	133072	S
	133034	R
	133068	R
*S. warneri*	133029	S
	133019	R
*S. hominis*	133075	S
	133097	R
*S. cohnii*	133089	S
	133041	R
*S. capitis*	133095	S
	133092	R
*S. simulans*	133080	S
	133053	R
*S. lugdunensis*	133074	S
	133082	R
*S. saprophyticus*	133002	S
	133091	Unknown

**Table 4:** List of Staphylococcal isolates used in mixed bacterial culture experiments.

Staphylococcal Species	Cambridge Identity Codes (CC)	Methicillin Sensitivity Profile
*S. aureus*	88048	R
	17046	R
	100085	S
*S. epidermidis*	72020	R
*S. haemolyticus*	133034	R

## Data Availability

Data is contained within the article.
